# Supplementary material for: Gut Microbiota Linked with Reduced Fear of Humans in Red Junglefowl Has Implications for Early Domestication
Source: Adv Genet (Hoboken). 2021 Dec 9;2(4):2100018. doi: 10.1002/ggn2.202100018 (PMC9744516; doi:10.1002/ggn2.202100018)
Supplement: Supplementary file 1 — Supporting Information [file GGN2-2-2100018-s004.pdf]

Supporting Information

Bacterial 16S metabarcoding methods

The initial PCR mixture amplifying community bacterial DNA used 1X AccuPrime SuperMix II (Invitrogen, Eugene, OR, USA), 0.5 μM (each) forward and reverse primers, ~5 ng DNA , and DNase/RNase-free water to a final volume of 20 μL. The PCR profile consisted of an initial denaturation step at 95°C for 2 min, followed by 30 cycles of 95°C for 15 s, 55°C for 15 s, and 68°C for 40 s and a final elongation step at 68°C for 4 min.

Dual indices and sequencing adapters were attached to the amplicons in an additional PCR reaction using a Nextera XT v2 index kit (Illumina Inc.). Five microliters of the first PCR product, 12 μL AccuPrime SuperMix II (Invitrogen, Eugene, OR, USA), 2 μL of each Nextera i7 and i5 index primer, and DNase/RNase-free water were mixed to a final volume of 28 μL in the second PCR. The PCRII profile consisted of an initial denaturation step at 95°C for 2 min, followed by 5-7 cycles of 95°C for 10 s, 55°C for 20 s, and 68°C for 20 s and a final elongation step at 68°C for 5 min.

Prior to sequencing, the final PCR products were visualized on a 2% agarose gel electrophoresis, pooled equimolar (or of each blank at the volume of the least concentrated sample), and purified with a 0.7X ratio of Agencourt AMPure bead purification (Beckman Coulter, Inc, Brea, California), following the manufacturer's protocol. Final concentrations were measured on a Qubit™ and library quality was verified on the 2200 TapeStation prior to sequencing on an Illumina MiSeq platform using 250PE chemistry.

Supplementary Figures:

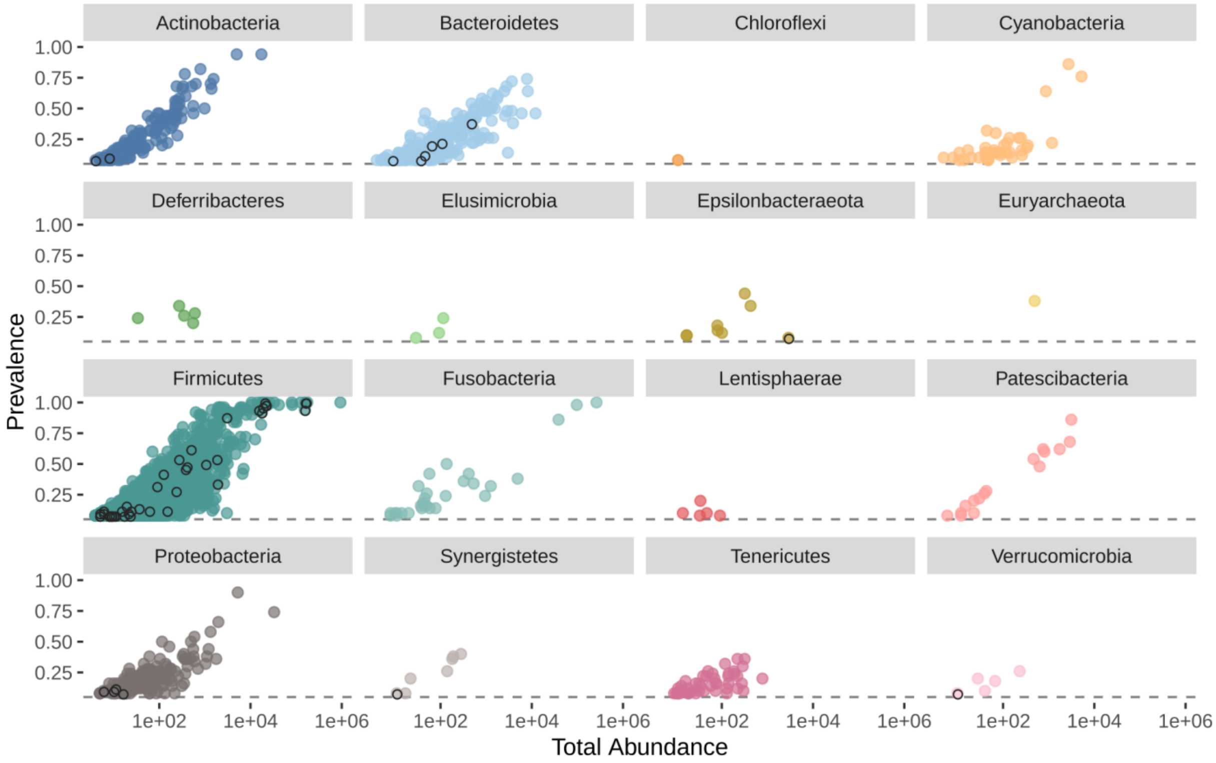

Suppl. Fig. 1. Differential abundance of gut-associated bacteria between behavioural phenotypic groups of red junglefowl occur in both rare and abundant taxa. Prevalence plots of 2582 bacterial 16S rRNA amplicon sequence variants (ASVs) found in red junglefowl faecal samples at the phylum level. Each point represents the total counts of a unique ASV corresponding to the fraction of individuals it was detected in. ASVs that were differentially abundant (n=57) or discriminant (n=50) in either the high or low fear towards human group are highlighted with open black circles.

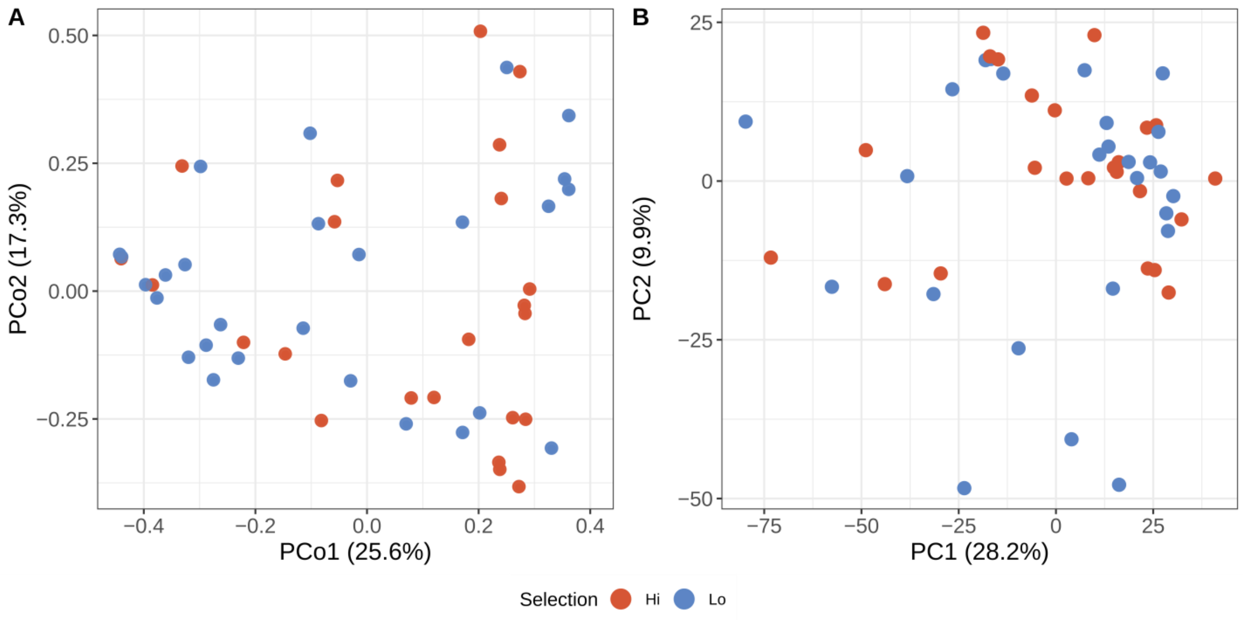

Suppl. Fig. 2. Ordination of the gut microbial communities of the red junglefowl highlighted by behavioral selection line. A) Principle coordinate analysis (PCoA) of the Bray-Curtis dissimilarity matrices of the relative abundance of bacterial 16S ASV sequence data. **B)** Principle component analysis of the log-ratios of the number of reads mapped to each of the MAGs per RJF sample. Both ordination plots show little clustering and a lack of grouping based on behavioral selection line.

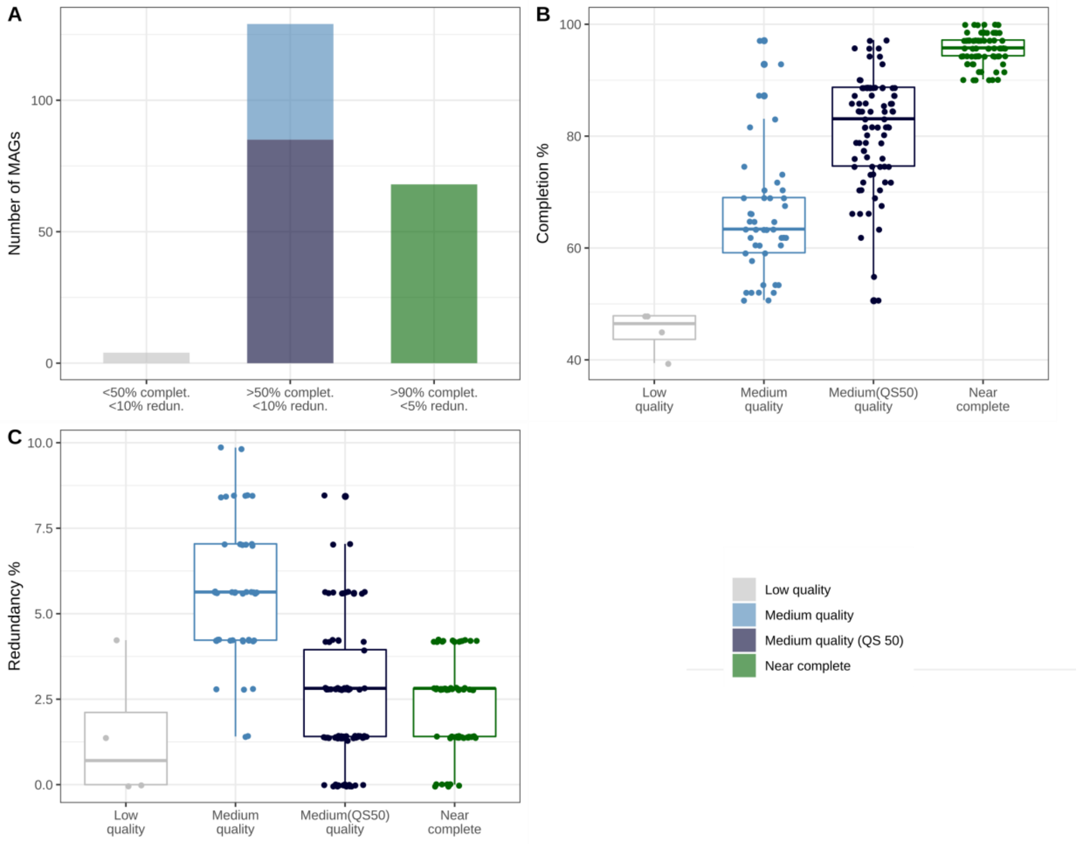

Suppl. Fig. 3. Quality metrics estimated by Anvi'o for the 198 bins generated by a manual binning strategy within the anvi'o framework. Low quality BINs were removed from the analyses. **A)** Number of bins recovered according to the level of genome completeness and redundancy. Box and whisker plots for the **B)** percentage of completion and **C)** percentage of redundancy for each bin grouped by the quality standard of the draft genome. QS = completeness – (5 × contamination).

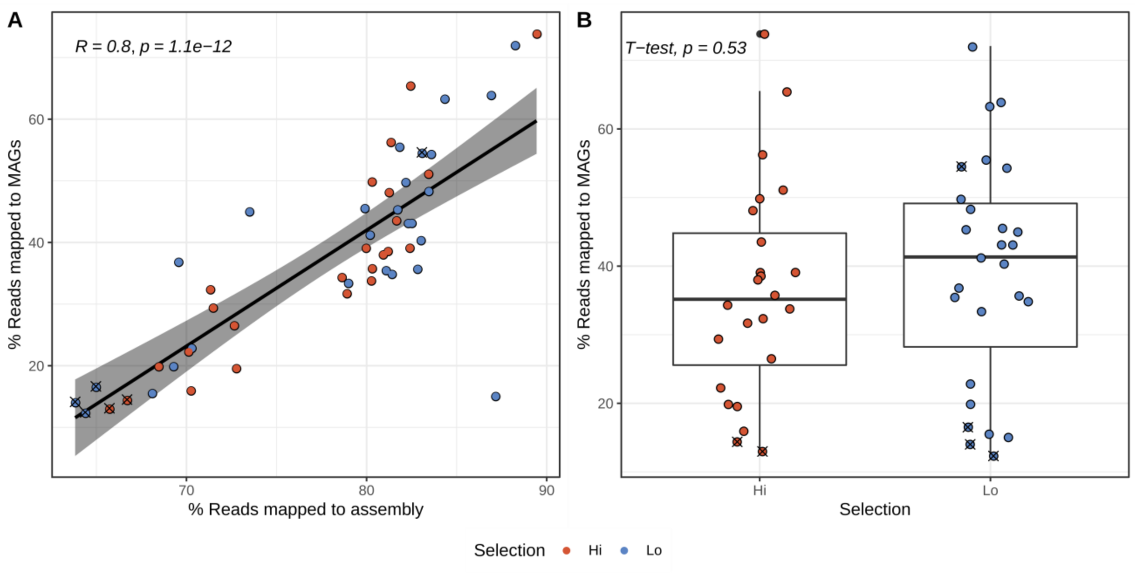

Suppl. Fig. 4. The percent of reads that map to the MAGs is correlated with the quality of the assembly and the sequencing platform facility but equally represent each behavioral phenotype. **A)** The percent of reads that mapped to the collection of MAGs out of the total number of reads, excluding reads that mapped to the chicken and human genome is presented for each of the high fear (orange) or low fear (blue) red junglefowl fecal metagenomes as a function of the percent of reads that mapped to all contigs in the assembly. Black curve represents a linear regression model with the grey shaded area marking the 95% confidence intervals. R-squared value and p-value for the linear regression appear above the curve. **B)** Box and whisker plots for the percentage of reads that map to the raw assembly contigs for high fear (orange) and low fear (blue) red junglefowl fecal metagenomes. X = samples with a sequencing depth of less than 10 million single-end reads (<1 Gb) after quality control and host filtering and were removed from downstream shotgun sequence analyses.
